# Physical Activity, Metabolic Dysfunction, and the Kynurenine Pathway in Endometriosis and Polycystic Ovary Syndrome: A Literature Review

**DOI:** 10.3390/biom16030440

**Published:** 2026-03-15

**Authors:** Noémi Varga, Rita Kis-György, Lilla Ajkay-Donáth, Zoltán István Tapody, Evelin Vágvölgyi-Sümegi, Tamás Körtési, Gábor Nagy-Grócz

**Affiliations:** 1 Department of Theoretical Health Sciences and Health Management, Faculty of Health Sciences and Social Studies, University of Szeged, H-6726 Szeged, Hungary; noemi88varga@gmail.com (N.V.); donathlilla@gmail.com (L.A.-D.); h162152@gmail.com (Z.I.T.); vagvolgyi-sumegi.evelin@szte.hu (E.V.-S.); kortesi.tamas@szte.hu (T.K.); 2 Doctoral School of Experimental and Preventive Medicine, University of Szeged, H-6725 Szeged, Hungary; kis-gyorgy.rita@szte.hu; 3 Section of Health Behaviour and Health Promotion, Faculty of Health Sciences and Social Studies, University of Szeged, H-6726 Szeged, Hungary; 4 HUN-REN-SZTE Neuroscience Research Group, Hungarian Research Network, Danube Neuroscience Research Laboratory, University of Szeged, H-6725 Szeged, Hungary; 5 Preventive Health Sciences Research Group, Incubation Competence Centre, Centre of Excellence for Interdisciplinary Research, Development and Innovation, University of Szeged, H-6720 Szeged, Hungary

**Keywords:** endometriosis, polycystic ovary syndrome (PCOS), insulin resistance, physical activity, exercise intervention, kynurenine pathway

## Abstract

Endometriosis and PCOS are both leading causes of female infertility, each affecting approximately 10% of reproductive-aged women worldwide. Both conditions markedly impair quality of life by affecting physical health, emotional well-being, mental health, and social functioning, and they impose a substantial economic burden through surgical treatments, assisted reproductive technologies, and work absenteeism. Insulin resistance (IR) plays a key role in the pathogenesis of both disorders by promoting chronic low-grade inflammation and disrupting sex hormone homeostasis. Consequently, interventions targeting metabolic dysfunction and inflammatory processes may improve clinical outcomes. In this context, the kynurenine system—the primary metabolic pathway of tryptophan degradation—has emerged as a potential mechanistic link between inflammation, metabolic disturbances, and reproductive disorders. Chronic inflammation and psychological stress can enhance kynurenine pathway activation, leading to immune dysregulation, oxidative stress, altered neuroendocrine signaling, and impaired ovarian function. Dysregulated kynurenine metabolism has also been associated with IR and mood disturbances, which are common features of endometriosis and PCOS. Physical activity (PA) is widely recognized for its anti-inflammatory, metabolic, and stress-reducing effects. Emerging evidence suggests that PA may also modulate the kynurenine system by shifting tryptophan metabolism toward neuroprotective pathways. Various exercise modalities—including aerobic, resistance, and mind–body exercises—have shown beneficial effects; however, well-designed long-term studies are still needed. The aim of this review is to synthesize and critically evaluate the published literature on the effects of PA on IR, inflammation, kynurenine metabolism, and reproductive health in women with endometriosis and PCOS.

## 1. Introduction

Endometriosis and polycystic ovary syndrome (PCOS) are prevalent causes of female infertility worldwide, each affecting approximately 10% of women of reproductive age. [1]. Both conditions are closely linked to metabolic disturbances, including hyperinsulinemia and insulin resistance (IR) [2,3], as well as low-grade chronic inflammation [4,5], which exacerbate symptom severity and impair ovarian function. Insulin, beyond its classical role in glucose homeostasis, influences lipid metabolism, protein synthesis, cell proliferation [6], and ovarian steroidogenesis [7], while chronic hyperinsulinemia can trigger receptor downregulation and tissue-specific IR [8]. Similarly, persistent low-grade inflammation, promoted by lifestyle factors and visceral adiposity, further impairs metabolic and reproductive functions [9,10].

An emerging mechanistic link between metabolic dysfunction, inflammation, and reproductive health is the kynurenine pathway (KP) [11,12,13,14], the primary pathway of tryptophan (Trp) degradation. Chronic inflammation and stress can upregulate key enzymes of the KP in humans [15], resulting in increased production of bioactive metabolites that modulate immune regulation, oxidative stress, and neuroendocrine signaling. Dysregulated kynurenine metabolism has been associated with IR [16], impaired ovarian function [17], and mood disturbances [18,19], all of which are commonly observed in women with endometriosis and PCOS. Moreover, an imbalance between neurotoxic and neuroprotective kynurenine metabolites may contribute to both somatic symptoms and psychosocial burden in these conditions.

The pathophysiology of endometriosis is multifactorial and involves estrogen-dependent ectopic endometrial growth [20], immune dysregulation [21], and a chronic inflammatory microenvironment [22], all of which contribute to lesion establishment and symptom progression. PCOS, in contrast, is primarily characterized by hyperandrogenism [23], ovarian dysfunction [23], and metabolic disturbances, including IR in adipose tissue [24], which can occur even in non-obese individuals and plays a central role in the syndrome’s systemic manifestations. Importantly, both conditions exhibit significant immunometabolic alterations: IR, chronic low-grade inflammation, and dysregulation of the KP may interact in a bidirectional or self-reinforcing manner, potentially forming a vicious cycle that contributes to disease initiation, progression, and symptom persistence.

Physical activity (PA) has emerged as a multifaceted intervention capable of improving insulin sensitivity, modulating inflammatory responses, and regulating hormonal profiles. Importantly, PA may also beneficially influence KP by shifting Trp metabolism toward anti-inflammatory and neuroprotective pathways [25]. Evidence suggests that structured exercise reduces pelvic pain, improves quality of life, and attenuates androgenic and metabolic dysfunctions in endometriosis and PCOS [26,27].

This work is a narrative review that synthesizes and critically evaluates the published literature on the effects of PA on kynurenine metabolism and reproductive health in women with endometriosis and PCOS. Based on the available evidence, it highlights potential mechanistic pathways and emphasizes the need for long-term, supervised exercise interventions to improve symptom management, metabolic function, and overall quality of life. We conducted a literature search in the PubMed database, reviewing publications published between 1988 and 2026. The following keywords were used: endometriosis, PCOS, PA, kynurenine system, IR, and physical intervention. The search was restricted to articles published in English. Initially, abstracts were screened for relevance, after which full-text articles were assessed according to the predefined inclusion criteria. Earlier publications were primarily used to describe the pathophysiological background of the diseases, while more recent studies were examined with particular attention to the potential relationship between the KP and PA in the context of these two conditions.

## 2. Endometriosis and Polycystic Ovary Syndrome: Clinical Features and Metabolic–Inflammatory Mechanisms

Endometriosis is a chronic gynecological disorder most commonly characterized by dysmenorrhea; however, chronic pelvic pain of variable intensity and frequency is also prevalent [28]. Depending on the anatomical location, extent, and inflammatory activity of endometriotic lesions, patients may experience dysuria, dyschezia, dyspareunia, or, in severe cases, hematuria or rectal bleeding [29]. Notably, infertility may represent the sole clinical manifestation in a subset of affected women [30]. Importantly, clinical presentation is highly heterogeneous and closely related to disease stage and lesion subtype (e.g., superficial peritoneal lesions, ovarian endometrioma, or deep infiltrating endometriosis), which differ in inflammatory activity, fibrosis, and neuroangiogenic profile. This heterogeneity has significant implications not only for symptom burden but also for systemic metabolic and immunological alterations observed across studies. Although the pathogenesis of endometriosis remains incompletely understood, several mechanisms have been implicated, including retrograde menstruation [31], immune dysfunction, and chronic inflammation [32] driven in part by adipokines such as leptin [33]. Inflammation plays a fundamental role in endometriosis pathophysiology by driving tissue remodeling, lesion formation, fibrosis, and clinical manifestations [34], such as pain and infertility, through impaired oocyte quality, reduced ovarian reserve, and diminished endometrial receptivity. Chronic inflammation also increases the risk of malignant transformation. In addition, proangiogenic mediators, including vascular endothelial growth factor (VEGF), interleukin (IL)-1β, and tumor necrosis factor (TNF)-α, promote lesion vascularization and persistence [35]. Furthermore, common comorbidities such as irritable bowel syndrome, mood disorders, and chronic pain syndromes—each independently associated with altered inflammatory and Trp metabolism—may confound the interpretation of circulating biomarkers if not adequately controlled for.

Recent studies have identified a significant association between the triglyceride-glucose index—a reliable surrogate marker of IR—and the risk of endometriosis [36,37], suggesting a potential role for metabolic dysfunction in disease development. Endometriosis is consistently associated with elevated estrogen levels, and prolonged estrogen receptor activation by endogenous or exogenous estrogens [38] may promote excessive insulin secretion, pancreatic β-cell dysfunction, and peripheral IR. IR, in turn, may induce a chronic inflammatory state, establishing a self-perpetuating cycle that disrupts endometrial tissue homeostasis and facilitates the attachment, invasion, and spread of ectopic endometrial cells [9,39]. Inflammation related to IR may further impair endothelial function, thereby promoting aberrant angiogenesis and lesion growth [40] while also altering the balance between cell proliferation and apoptosis [41]. However, reported associations between IR-related indices and endometriosis are not entirely consistent, likely reflecting variability in disease stage distribution, lesion subtype predominance, BMI composition, and differences in metabolic phenotyping across studies. Stratified analyses according to disease severity and metabolic status remain limited but are essential for clarifying these relationships.

Lifestyle factors play a critical role in modulating endometriosis risk. Diet and lifestyle interventions influence estrogen metabolism, inflammatory pathways, menstrual cyclicity, and prostaglandin synthesis. Diets rich in green vegetables, fruits, and omega-3 and omega-6 polyunsaturated fatty acids have been associated with a reduced risk of endometriosis [42], whereas high intake of trans fats and red meat [43,44] has been linked to increased disease risk. Nonetheless, dietary studies are frequently limited by heterogeneity in case definition, staging, and adjustment for confounders such as BMI, PA, and comorbid gastrointestinal or mood disorders, all of which may independently influence inflammatory and metabolic parameters.

PCOS is a heterogeneous endocrine–metabolic disorder characterized by a broad spectrum of clinical manifestations, not all of which are present in every patient [23]. Core features include menstrual irregularities such as oligomenorrhea or amenorrhea, chronic anovulation, infertility, hyperandrogenic symptoms (hirsutism, acne, alopecia), visceral adiposity, acanthosis nigricans, and, in some cases, pelvic pain [23]. According to the Rotterdam criteria, distinct phenotypes (A–D) can be identified based on the presence or absence of hyperandrogenism, ovulatory dysfunction, and polycystic ovarian morphology, and these phenotypes differ substantially in metabolic risk. Hyperandrogenic phenotypes generally display a more adverse metabolic and inflammatory profile than non-hyperandrogenic forms, underscoring the importance of phenotypic stratification in mechanistic and biomarker studies. Compared with BMI-matched controls, women with PCOS—including those classified as normal weight—exhibit increased intra-abdominal adiposity and a higher whole-body fat-to-lean mass ratio [45]. These alterations are closely associated with whole-body IR, hyperinsulinemia, and elevated circulating androgen levels [46,47]. Importantly, lean and obese PCOS phenotypes differ significantly in the degree of IR and inflammatory activation, and pooling these subgroups without stratification may obscure clinically meaningful metabolic differences. Across the BMI spectrum, PCOS is characterized by adipocyte dysfunction, reflected by abnormal adipocyte size distribution and disrupted adipokine secretion [48,49]. Excess visceral fat promotes free fatty acid release, ectopic lipid deposition, and a proinflammatory cytokine milieu, collectively impairing insulin signaling [49,50,51]. Circulating adiponectin levels—an insulin-sensitizing adipokine—are markedly reduced even in lean women with PCOS [52], while decreased glucose transporter type 4 (GLUT4) expression [53] and impaired glycogen synthesis [54] further compromise glucose uptake in adipose tissue.

Hepatic and skeletal muscle IR further contribute to the metabolic phenotype of PCOS. Hepatic steatosis and altered hepatokine secretion exacerbate hepatic IR through increased intracellular lipid accumulation and disrupted insulin signaling [50,55]. Skeletal muscle insulin sensitivity is also reduced, accompanied by increased intramuscular lipid content and alterations in muscle fiber composition [52,56]. However, the extent of hepatic and muscular involvement varies widely across studies, partly due to differences in imaging modalities, IR assessment methods, and inclusion criteria regarding BMI and medication use. At the ovarian level, hyperandrogenism represents a central feature of PCOS and remains highly sensitive to insulin. Insulin directly stimulates ovarian theca cells by enhancing androgen biosynthetic pathways, with PCOS theca cells exhibiting exaggerated insulin-stimulated testosterone production compared with controls [57,58]. Hyperinsulinemia further amplifies luteinizing hormone (LH) responsiveness, suppresses insulin-like growth factor binding protein-1 (IGFBP-1), increases bioavailable insulin-like growth factor-1 (IGF-1), and reduces sex hormone-binding globulin (SHBG) levels [59], thereby intensifying ovarian androgen production. Notably, androgens themselves may promote insulin hypersecretion, reinforcing this pathological feedback loop. Pharmacological exposures—including combined oral contraceptives, metformin, antiandrogens, and insulin-sensitizing agents—can substantially modify these hormonal and metabolic interactions. Failure to account for current or recent medication use represents a major source of heterogeneity in clinical and biomarker studies. In addition, methodological factors may contribute to conflicting findings in metabolic and inflammatory research in PCOS. Variability in fasting status, menstrual cycle phase (particularly challenging in anovulatory women), circadian timing of sampling, and acute stress exposure may influence circulating metabolic and inflammatory markers. Without rigorous standardization and detailed reporting, distinguishing true biological variability from methodological noise remains difficult. Management strategies for PCOS, therefore, emphasize weight reduction where appropriate, dietary modification to reduce inflammation and visceral adiposity, and regular PA to improve insulin sensitivity and metabolic health.

**Figure 1:**
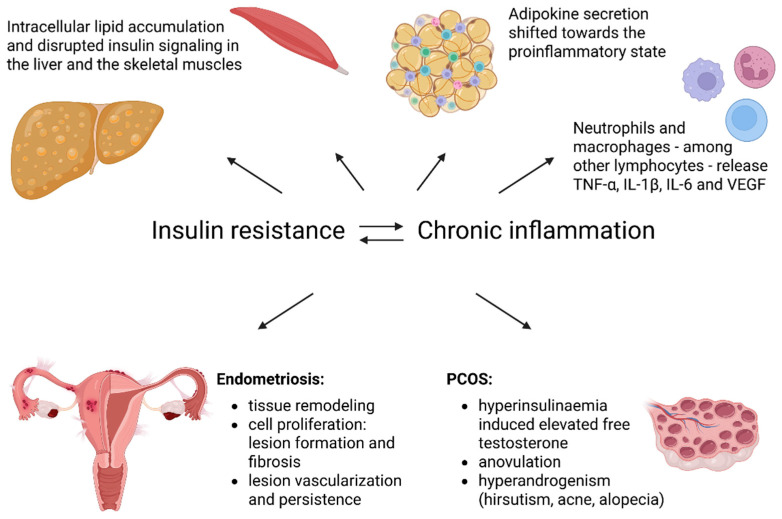
The effects of IR and chronic inflammation on different organs and tissues regarding the pathogenesis and exacerbation of endometriosis and PCOS. Abbreviations: IL-1β—interleukin-1β; IL-6—interleukin-6; PCOS—polycystic ovary syndrome; TNF-α—tumor necrosis factor-α; VEGF—vascular endothelial growth factor. Created in BioRender. Varga, N. (2026). BioRender.com/tsiul3p.

Taken together, endometriosis and PCOS are complex, multifactorial disorders involving tightly interconnected inflammatory, hormonal, and metabolic pathways. Their pathophysiology is driven by self-reinforcing feedback loops between estrogen imbalance, IR, adipose tissue dysfunction, and chronic inflammation, resulting in highly heterogeneous clinical manifestations, as illustrated in Figure 1. A critical interpretation of the literature requires careful consideration of phenotypic diversity, disease stage, metabolic status, comorbidities, medication exposure, and methodological variability, all of which substantially influence reported associations and may account for apparently conflicting findings across studies.

## 3. Insulin Beyond Glucose Homeostasis: Hyperinsulinemia and Inflammation

Insulin, secreted by the β-cells of the pancreas, is a key metabolic hormone that regulates numerous processes throughout the body. Beyond its central role in glucose homeostasis, insulin influences lipid metabolism and protein synthesis, cell growth and proliferation, stress responses, and autophagy, as well as—most relevant to the present context—ovarian steroidogenesis and folliculogenesis [6,60]. Owing to its multifaceted actions, insulin secretion is tightly regulated by a wide range of stimuli, including nutrients (glucose, fatty acids, and amino acids), other hormones, paracrine and autocrine factors, and neurotransmitters [61,62].

It is important to note that, with regard to dietary intake, attention must be paid to both the quantity and timing of all macronutrients—not carbohydrates alone—in order to prevent hyperinsulinemia and IR [63,64]. Moreover, these conditions have also been documented in individuals with normal or only mildly elevated body weight, underscoring that visceral adipose tissue accumulation is a key driver of IR, while obesity is more accurately viewed as a consequence and, in the long term, an aggravating factor [65,66,67].

Hyperinsulinemia may arise from β-cell proliferation or from chronically heightened β-cell responsiveness to nutrient stimulation as a result of persistent overnutrition. Frequent consumption of diets rich in sugars and fats induces exaggerated postprandial insulin secretion [68], which, over time, can impair the regulation of basal insulin release. This dysregulation ultimately leads to elevated fasting insulin levels and persistent hyperinsulinemia even in the absence of nutrient stimulation. Additional contributors to fasting hyperinsulinemia include increased production of reactive oxygen species in response to inflammation, as well as excess long-chain acyl-CoA esters, both of which disrupt normal β-cell function [69]. At the cellular level, it is well established that chronic hypersecretion of a signaling molecule eventually leads to desensitization of its signaling pathway, primarily through receptor downregulation. In the case of insulin, hyperinsulinemia promotes insulin receptor degradation and reduces insulin receptor mRNA expression [70], thereby inducing IR.

Clinically, IR is defined as a state in which an excessive amount of insulin is required to facilitate glucose uptake into target cells, or in which blood glucose concentrations remain higher than expected despite normal circulating insulin levels. This definition, however, focuses predominantly on glucose homeostasis and does not fully capture the broad spectrum of insulin-mediated cellular processes. Importantly, several insulin-dependent signaling pathways appear to remain sensitive to insulin despite the presence of IR, resulting in the upregulation of downstream biochemical processes, some of which contribute to the pathogenesis of PCOS [2,71] and the progression and worsening of endometriosis [72].

Persistent inflammation, driven by continuous inflammatory stimuli or by failure to achieve resolution, can evolve into systemic low-grade chronic inflammation—a subtle form of the inflammatory response that remains below the threshold for overt clinical symptoms. This chronic inflammatory state represents a common pathophysiological hallmark of most non-communicable diseases [73]. Sustained elevation of proinflammatory cytokines, including TNF-α, IL-1, and IL-6, together with increased expression of adhesion molecules, promotes progressive tissue damage and, over time, contributes to the development of a wide spectrum of disorders, including cardiovascular and metabolic diseases, as well as cancer [74,75,76,77,78].

Among the various triggers of chronic low-grade inflammation, lifestyle-related factors play a central role. Key contributors include smoking; consumption of energy-dense but nutritionally poor diets—characterized by high intakes of ultra-processed foods, saturated and trans fats, and refined sugars, alongside low intakes of fiber, protein, complex carbohydrates, vitamins, and minerals; excessive alcohol consumption; inadequate stress management; poor sleep quality; and sedentary behavior [78,79,80,81]. Sedentary behavior further promotes the accumulation of visceral adipose tissue, which secretes proinflammatory adipokines such as leptin and resistin, while reducing the release of anti-inflammatory and insulin-sensitizing adipokines, most notably adiponectin, as well as other anti-inflammatory cytokines [81,82,83]. Collectively, these alterations exacerbate systemic inflammation and contribute to the development of IR.

**Figure 2:**
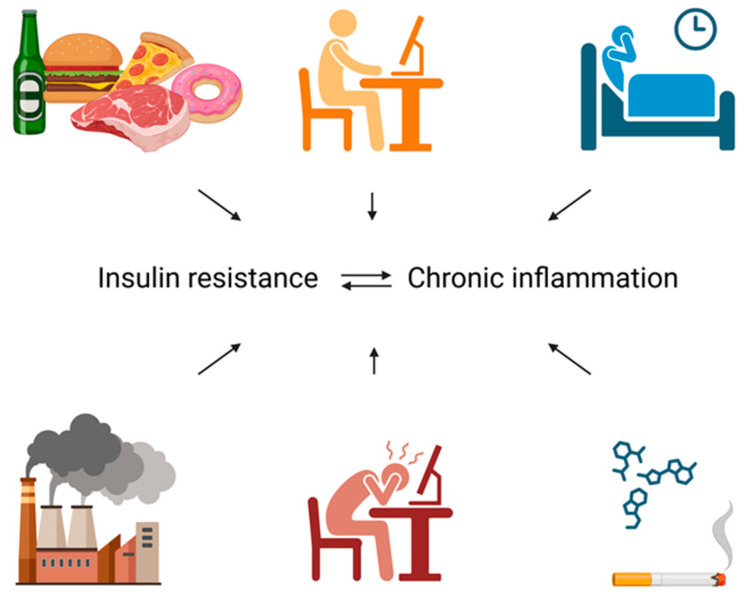
Lifestyle and environmental factors contributing to IR and chronic inflammation. Created in BioRender. Varga, N. (2026). BioRender.com/g4eqnn2.

In summary, insulin dysregulation plays a central role at the interface of metabolic, inflammatory, and reproductive processes. Chronic hyperinsulinemia and IR—driven by visceral adiposity and lifestyle factors—promote low-grade systemic inflammation and selectively activate pathogenic signaling pathways implicated in PCOS and endometriosis, as illustrated in Figure 2.

## 4. The Kynurenine Pathway

**Figure 3:**
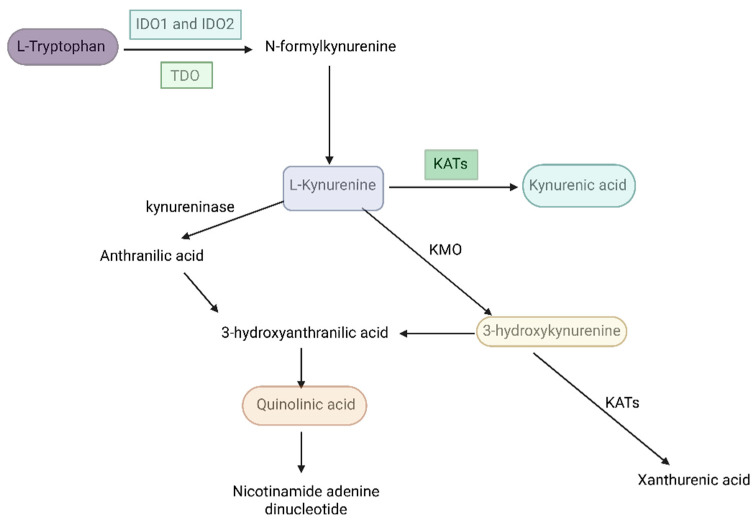
The KP. The colored background highlights key KP metabolites that may play a role in endometriosis and PCOS. Abbreviations: IDO1 and IDO2—indoleamine 2,3-dioxygenase 1 and 2; KATs—kynurenine aminotransferases; KMO—kynurenine 3-monooxygenase; TDO—tryptophan 2,3-dioxygenase. Created in BioRender. Varga, N. (2026). BioRender.com/c6kclrt.

Trp is an essential amino acid with a central role in neurobiological and metabolic regulation. While Trp is widely recognized as the precursor of serotonin, only a minor fraction of dietary Trp is utilized for serotonin synthesis. In contrast, the vast majority of Trp in mammalian cells is catabolized through the KP (Figure 3), which represents the principal route of Trp degradation and a critical interface between metabolism, immune regulation, and neuroendocrine function [84,85].

Emerging evidence indicates that gut microbiota composition can influence systemic Trp availability and metabolism, thereby linking dietary factors and intestinal homeostasis to downstream KP activity and gastrointestinal function [86,87].

The KP is initiated by the conversion of L-Trp to L-kynurenine (L-KYN) via the rate-limiting enzymes indoleamine 2,3-dioxygenase (IDO1 and IDO2) and tryptophan 2,3-dioxygenase (TDO). Importantly, the activity of these enzymes is highly sensitive to inflammatory mediators, stress hormones, and metabolic cues, positioning the KP as a possibly responsive metabolic sensor in states of chronic low-grade inflammation and IR [11,88]. From L-KYN, multiple downstream metabolites are generated through distinct enzymatic branches, giving rise to compounds with markedly different—and sometimes opposing—biological effects.

One such metabolite, kynurenic acid (KYNA), is produced via kynurenine aminotransferases (KATs) and acts as an endogenous antagonist of excitatory glutamatergic signaling [89,90], conferring neuroprotective and neuromodulatory properties [91,92]. In contrast, alternative branches of the pathway yield metabolites such as 3-hydroxykynurenine (3-HK) and quinolinic acid (QUIN), which are associated with oxidative stress, mitochondrial dysfunction, and excitotoxicity [93,94,95].

Notably, the terminal steps of the KP lead to the synthesis of nicotinamide adenine dinucleotide (NAD^+^), a key cofactor in mitochondrial energy production and redox balance. Dysregulation of this branch may therefore directly impair cellular energy metabolism, linking altered Trp degradation to mitochondrial dysfunction [96] observed in metabolic and inflammatory diseases. Consistent with this, aberrant kynurenine metabolism has been implicated in a wide range of neurological, psychiatric, and metabolic conditions [97,98], including disorders characterized by chronic inflammation, IR, and stress-related neuroendocrine alterations. Within this broader framework, the KP emerges as a mechanistic link connecting lifestyle-related factors, immune activation, metabolic dysfunction, and brain–body communication. Given its sensitivity to inflammation, stress, and metabolic status, modulation of the KP—such as through PA [99,100,101] and dietary interventions [102,103]—may represent a biologically plausible pathway through which lifestyle factors influence metabolic, immune, and neuroendocrine outcomes.

The association between the KP and endometriosis or PCOS appears to be primarily mediated through chronic inflammation, with reports indicating an upregulation of the pathway that leads to reduced Trp availability and a shift in the balance between neurotoxic and neuroprotective metabolites [17,104]. Elevated L-KYN-to-Trp ratios, a surrogate marker of IDO activity [105], have been associated with systemic inflammation [104], IR [106], and depressive symptoms [107]—all of which are common features in both disorders [11].

Kynurenines exert their biological effects through multiple receptor systems. Their neuroactive actions are most often discussed in relation to the glutamatergic N-methyl-D-aspartate (NMDA) receptor, particularly in the context of excitotoxicity and pain sensitization. However, KP metabolites act on several additional targets. One of the most relevant among these is the aryl hydrocarbon receptor (AhR), for which L-KYN serves as an endogenous ligand. AhR is a ligand-activated transcription factor that is widely expressed in immune, endocrine, and reproductive tissues [108]. Upon activation, it translocates to the nucleus and regulates the expression of genes involved in immune regulation, inflammatory processes, and cellular differentiation. In this way, AhR integrates metabolic and immune signals and links them to broader physiological responses. Importantly, the downstream effects of AhR activation appear to be highly context-dependent and may differ according to ligand type, tissue environment, and inflammatory status [109].

Increasing evidence suggests that AhR signaling is relevant in gynecological disorders characterized by chronic inflammation and hormonal imbalance. In endometriosis, AhR activation may support the survival of ectopic lesions by promoting local immune tolerance. This involves reduced effector T-cell activity and increased regulatory T-cell differentiation. AhR also interacts with estrogen receptor signaling and angiogenic pathways, including the regulation of vascular endothelial growth factor expression, which may further facilitate lesion growth and vascularization [110]. In PCOS, altered kynurenine–AhR signaling has been associated with low-grade inflammation, IR, and adipose tissue dysfunction. However, findings on circulating kynurenine levels in PCOS are inconsistent, with some studies reporting elevations while others show no significant differences, likely reflecting phenotypic heterogeneity and methodological variations. These processes are closely linked to ovarian hyperandrogenism and impaired folliculogenesis. Taken together, dysregulation of kynurenine–AhR signaling may represent a shared immunoendocrine mechanism connecting inflammation, metabolic disturbances, and reproductive dysfunction in both endometriosis and PCOS [104].

## 5. The Potential Role of Physical Activity in the Metabolic, Inflammatory, and Hormonal Regulation of PCOS and Endometriosis, with a Particular Focus on the KP

PA has gained increasing recognition as a complex, multimodal intervention capable of influencing metabolic, inflammatory, endocrine, and neuroimmune pathways. In the context of endometriosis and PCOS, PA represents a particularly promising non-pharmacological therapeutic strategy. Importantly, however, the strength of clinical evidence differs between the two conditions: interventional data are substantially more robust in PCOS, whereas studies in endometriosis remain comparatively fewer and often smaller in scale.

As detailed earlier, impaired insulin sensitivity represents a central pathophysiological feature of PCOS and is increasingly recognized in endometriosis as well. Regular PA, especially aerobic training, has been shown to improve insulin sensitivity via both insulin-dependent and insulin-independent pathways [111,112]. Skeletal muscle contractions stimulate glucose uptake via translocation of GLUT4 to the cell membrane, mediated by activation of AMP-activated protein kinase and calcium–calmodulin-dependent pathways [113]. Clinical trials and meta-analyses—predominantly conducted in women with PCOS—have demonstrated that aerobic exercise, resistance training, and combined exercise programs significantly reduce fasting insulin levels, homeostatic model assessment of IR (HOMA-IR), and postprandial glucose excursions [114,115,116]. Importantly, these benefits are observed even in the absence of significant weight loss, highlighting exercise-induced metabolic adaptations independent of adiposity reduction. In contrast, although improvements in metabolic parameters have been suggested in women with endometriosis engaging in regular PA, direct interventional evidence targeting IR in this population remains limited. Variability in disease stage, lesion subtype, and baseline metabolic status likely contributes to heterogeneous findings, underscoring the need for metabolically stratified exercise trials in endometriosis.

As we have previously detailed, chronic low-grade inflammation is a shared hallmark of endometriosis and PCOS, characterized by elevated circulating pro-inflammatory cytokines such as TNF-α, IL-6, and C-reactive protein (CRP) [32]. PA exerts potent immunomodulatory effects by reducing visceral adiposity, altering macrophage polarization, and inducing the release of anti-inflammatory myokines from contracting skeletal muscle [117]. In PCOS, moderate-intensity aerobic and combined exercise programs have consistently been shown to reduce systemic inflammatory markers, including CRP and TNF-α, while increasing anti-inflammatory cytokines such as IL-10 [118,119]. In endometriosis, available studies suggest improvements in inflammatory profiles and symptom burden, but the evidence base is smaller and more heterogeneous with respect to exercise modality and duration. Notably, the anti-inflammatory effects of exercise appear to follow a dose–response relationship. While moderate, structured exercise is protective, excessive or high-intensity unsupervised training may exacerbate inflammatory stress responses and hypothalamic–pituitary–adrenal axis (HPO) activation [120]. This consideration is particularly relevant in endometriosis, where pain flares and central sensitization may be triggered by overexertion, and in PCOS patients with severe obesity or deconditioning, in whom abrupt high-intensity programs may impair adherence and increase cardiometabolic risk. These observations emphasize the importance of individualized, supervised, and progressively structured exercise prescription.

Hormonal dysregulation is a defining feature of PCOS, particularly hyperandrogenism and altered gonadotropin secretion [23]. PA influences the HPO axis through improvements in insulin sensitivity, reductions in adipose-derived estrogen production, and modulation of SHBG levels [121,122]. In PCOS, structured aerobic and resistance training programs have been shown to increase SHBG levels and decrease circulating free testosterone [123,124,125]. These changes are clinically meaningful, as they correlate with improvements in menstrual regularity, ovulatory function, and hyperandrogenic symptoms such as hirsutism and acne [126].

In endometriosis, exercise may modulate estrogen dominance by reducing peripheral estrogen synthesis and enhancing estrogen metabolism toward less bioactive metabolites [127]. However, direct randomized controlled data specifically addressing endocrine endpoints in endometriosis are scarce. Most available studies focus on pain and quality-of-life outcomes rather than detailed hormonal profiling, highlighting an asymmetry in the evidence base compared with PCOS.

Pelvic pain is a major determinant of reduced quality of life in endometriosis [128] and is also reported by a subset of women with PCOS [129]. In PCOS, exercise interventions are consistently associated with improvements in psychological outcomes, including reduced anxiety and depressive symptoms, which are highly prevalent in this population [130]. PA has been shown to alleviate chronic pain through central and peripheral mechanisms, including enhanced endorphin release, improved pain modulation, and reduced central sensitization [131,132,133,134]. Randomized controlled trials indicate that yoga, aerobic exercise, and combined movement-based interventions significantly reduce pelvic pain intensity and improve health-related quality of life in women with endometriosis [135,136,137]. Mind–body modalities such as yoga may be particularly beneficial in endometriosis by integrating gentle movement with autonomic regulation and stress reduction, potentially mitigating pain flares.

From a practical perspective, different PA modalities may be preferentially tailored according to the dominant clinical features of each disorder. In PCOS, aerobic training (e.g., brisk walking, cycling) effectively targets IR and cardiometabolic risk; resistance training improves body composition and insulin sensitivity; and combined programs appear to confer additive benefits. In endometriosis, moderate-intensity aerobic exercise and low-impact resistance training may support anti-inflammatory and metabolic adaptations, while mind–body approaches (e.g., yoga, Pilates) may be especially useful for pain modulation and stress reduction. High-intensity interval training may offer metabolic advantages in selected PCOS patients under supervision, but caution is warranted in endometriosis and in individuals prone to symptom exacerbation.

**Figure 4:**
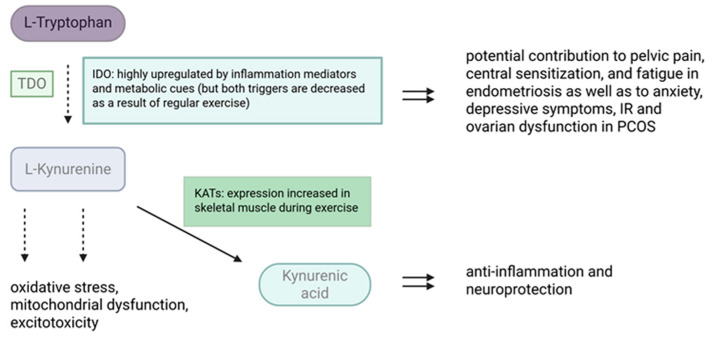
The initial steps of the KP and the enzymatic reactions influenced by exercise. The figure illustrates the main steps and effects of the KP starting from L-Trp. Inflammatory mediators activate the enzyme IDO, leading to increased production of L-KYN. L-KYN may contribute to oxidative stress, mitochondrial dysfunction, and excitotoxicity, and potentially to pelvic pain, central sensitization, and fatigue in endometriosis, as well as anxiety, depressive symptoms, IR, and ovarian dysfunction in PCOS. At the same time, increased KAT expression in skeletal muscle during exercise promotes the conversion of L-KYN to KYNA, which exerts anti-inflammatory and neuroprotective effects. Abbreviations: IDO—indoleamine 2,3-dioxygenase; IR—insulin resistance; KATs—kynurenine aminotransferases; PCOS—polycystic ovary syndrome. Solid arrows: direct steps in the KP; dashed arrows: multiple consecutive steps. Created in BioRender. Varga, N. (2026). BioRender.com/7bwh3fz.

Emerging evidence indicates that PA modulates Trp metabolism through the KP (Figure 4).

PA exerts a unique regulatory effect on the KP by promoting the conversion of L-KYN into KYNA within skeletal muscle [25]. Endurance and resistance training have been shown to increase the expression of KATs in humans [138,139], the enzymes responsible for converting L-KYN to KYNA. Both acute and chronic exercise elevate skeletal muscle KAT expression [101,139], thereby shifting kynurenine metabolism toward KYNA production. This shift may reduce the accumulation of neurotoxic metabolites and could have broader implications for energy homeostasis, inflammatory regulation, and neuroprotection.

In the context of endometriosis, local and systemic immune activation is likely to drive IDO-mediated Trp degradation [140], potentially contributing to pelvic pain, central sensitization, and fatigue. The capacity of PA to redirect kynurenine metabolism toward anti-inflammatory and neuroprotective pathways may help explain observed reductions in pain perception and improvements in quality of life among physically active women with endometriosis. However, direct interventional studies specifically quantifying kynurenine metabolites in exercising women with endometriosis are lacking.

Similarly, in PCOS, dysregulation of the KP may contribute to the high prevalence of anxiety and depressive symptoms, as well as to IR and ovarian dysfunction via inflammatory and oxidative mechanisms [17,104,141]. Exercise-induced normalization of kynurenine metabolism may therefore represent an important mechanistic link between PA and improvements in both metabolic and psychological outcomes in this population [142]. Although direct clinical data remain limited, emerging evidence from IR populations—including PCOS cohorts—suggests that structured lifestyle interventions incorporating exercise can reduce kynurenine levels and inflammatory markers.

**Figure 5:**
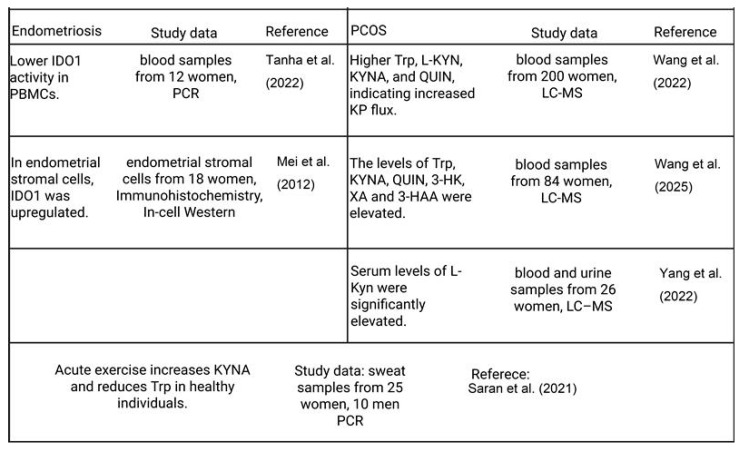
Summary of alterations in KP in endometriosis, PCOS, and in response to acute exercise. In endometriosis, reduced IDO1 activity in peripheral blood mononuclear cells (PBMC) [143] and upregulated IDO1 expression in endometrial stromal cells [144] have been reported. In PCOS, increased circulating levels of Trp and several KP metabolites (KYN, KYNA, QUIN, 3-HK, XA, and 3-HAA) indicate enhanced pathway flux [17,145,146]. Acute exercise in healthy individuals increases KYNA and decreases TRP levels [100]. Study characteristics (sample type, sample size, and analytical method) and corresponding references are indicated in the table. Abbreviations: 3-HAA—3-Hydroxyanthranilic; 3-HK—3-Hydroxykynurenine; IDO1—indoleamine 2,3-dioxygenase; KP—Kynurenine pathway; KYNA—kynurenic acid; LC-MS—liquid chromatography–mass spectrometry; L-KYN—L-kynurenine; PBMCs—peripheral blood mononuclear cells; PCOS—polycystic ovary syndrome; QUIN—quinolinic acid; Trp—tryptophan; XA—Xanthurenic acid. Created in BioRender. Varga, N. (2026). BioRender.com/ids967w.

Although preclinical and indirect lines of evidence are encouraging, human studies specifically examining how exercise modulates the KP in endometriosis and PCOS are still scarce (Figure 5). Future randomized controlled trials should clearly differentiate between disease phenotypes, apply standardized exercise protocols across aerobic, resistance, combined, and mind–body modalities, and incorporate metabolomic profiling of Trp and kynurenine metabolites to elucidate dose–response relationships and identify optimal, condition-specific exercise strategies.

## 6. Conclusions

Endometriosis and PCOS are complex, multifactorial disorders that share overlapping metabolic, inflammatory, endocrine, and neuroimmune disturbances despite their distinct clinical phenotypes. Accumulating evidence indicates that chronic low-grade inflammation, IR, hyperinsulinemia, altered adipokine signaling, and hormonal imbalance form interconnected pathophysiological networks that contribute to disease initiation, progression, and symptom burden in both conditions. Importantly, these processes are more strongly influenced by lifestyle factors than by genetic predisposition.

Insulin emerges as a central integrative mediator linking metabolic dysfunction to reproductive and inflammatory abnormalities. Persistent hyperinsulinemia not only drives IR but also amplifies ovarian androgen production in PCOS, disrupts endometrial homeostasis, promotes angiogenesis, and sustains inflammatory signaling in endometriosis. These effects are further reinforced by visceral adiposity, hepatic and skeletal muscle IR, and dysregulated adipokine and hepatokine secretion, establishing self-perpetuating pathogenic feedback loops.

Within this framework, the KP represents a critical immunometabolic interface connecting inflammation, IR, neuroendocrine regulation, and mitochondrial function. Chronic activation of the KP—driven by inflammatory and metabolic stressors—may contribute to systemic immune dysregulation, oxidative stress, mood disturbances, fatigue, and impaired reproductive function observed in both endometriosis and PCOS. Imbalances between neurotoxic and neuroprotective kynurenine metabolites further highlight the pathway’s dual role in disease pathophysiology.

PA emerges as a uniquely powerful, non-pharmacological intervention capable of targeting these interconnected mechanisms simultaneously. Through improvements in insulin sensitivity, reductions in visceral adiposity, modulation of inflammatory signaling, and normalization of hormonal profiles, regular structured exercise exerts broad systemic benefits. Notably, PA also induces a favorable shift in kynurenine metabolism by enhancing skeletal muscle-mediated conversion of L-KYN to KYNA, thereby reducing neurotoxic burden and promoting neuroprotection and immune balance. This mechanism provides a biologically plausible link between exercise, improved metabolic and psychological outcomes, and reduced pain perception. Despite growing evidence supporting the therapeutic potential of PA, substantial heterogeneity remains across existing studies, reflecting differences in exercise modality, intensity, duration, supervision, and participant characteristics. Moreover, direct human data examining PA modulation in endometriosis and PCOS remain limited. Future well-designed, long-term randomized controlled trials incorporating metabolomic, inflammatory, and endocrine profiling are therefore essential to define optimal exercise prescriptions and to clarify dose–response relationships.

In conclusion, endometriosis and PCOS should be conceptualized within an integrated immunometabolic framework, in which IR, chronic inflammation, and KP dysregulation may play central roles. PA represents a cornerstone intervention with the capacity to modulate these pathways concurrently, offering meaningful opportunities for symptom reduction, disease modification, and improved quality of life. Incorporating structured, individualized exercise programs into comprehensive management strategies may therefore be critical for addressing the systemic nature of these prevalent reproductive disorders.

## Data Availability

Data sharing is not applicable. No new data were created or analyzed in this study.

## Abbreviations

The following abbreviations are used in this manuscript:

3-HAA	3-Hydroxyanthranilic
3-HK	3-Hydroxykynurenine
AhR	Aryl hydrocarbon receptor
CRP	C-reactive protein
GLUT4	Glucose transporter type 4
HOMA-IR	Homeostatic Model Assessment of Insulin Resistance
HPO	Hypothalamic–pituitary–ovarian axis
IDO 1,2	Indoleamine 2,3-dioxygenase 1,2
IFN-γ	Interferon gamma
IGF-1	Insulin-like growth factor-1
IGFBP-1	Insulin-like growth factor-binding protein-1
IL	Interleukin
IR	Insulin resistance
KAT	Kynurenine aminotransferase
KMO	Kynurenine 3-monooxygenase
KP	Kynurenine pathway
KYNA	Kynurenic acid
LC-MS	Liquid chromatography–mass spectrometry
LH	luteinizing hormone
L-KYN	L-kynurenine
NAD^+^	Nicotinamide adenine dinucleotide
NF-κB	Nuclear factor kappa B
NMDA	N-methyl-D-aspartate
PA	Physical activity
PBMCs	Peripheral blood mononuclear cells
PCOS	Polycystic ovary syndrome
QUIN	Quinolinic acid
SHBG	Sex hormone–binding globulin
TDO	Tryptophan 2,3-dioxygenase
TNF-α	Tumor necrosis factor alpha
Trp	Tryptophan
VEGF	Vascular endothelial growth factor
XA	Xanthurenic acid
